# Digital transformation in higher education: logical framework, practical dilemmas, and implementation approaches

**DOI:** 10.3389/fpsyg.2025.1565591

**Published:** 2025-11-06

**Authors:** Juan Tang, Pin Huang, Shuangsheng Yan

**Affiliations:** School of Marxism, China Pharmaceutical University, Nanjing, China

**Keywords:** digital transformation, higher education, digital transformation in education, digital transformation in higher education, transformation models

## Abstract

The digital age comes with new demands and challenges for talent cultivation within the higher education system. The digital transformation in higher education has emerged as a critical element in addressing these challenges. This paper aims to develop an integrated qualitative model that advocates how digital transformation as a propelling force could be applied by university faculty and students during teaching and learning progress within the higher education institutions. An analysis of literature was performed to have a grasp of conceptual basis of digital transformation in higher education. Qualitative, in-depth case studies from Harvard University, Tsinghua University, and the University of Melbourne were engaged in to explore the transformation framework for university faculty and students. The study: 1) Proposes a triple logical framework comprising value logic, technological logic, and practical logic; 2) Examines four practical dilemmas encountered in advancing digital transformation within higher education teaching and learning environments based on logical framework, including both psychological and objective challenge for university teachers and students; 3) Outlines six implementation approaches to digital transformation in higher education, addressing aspects such as teaching concepts, teaching models, teaching resources, teaching scenarios, and evaluation systems. Finally, an illustrative digital SECI-based multimodal teaching model is suggested.

## 1 Introduction

Following thousands of years in the agrarian era and two hundred years in the industrial era, humanity has entered the information age and the digital era (Lei, 2024). The Fourth Industrial Revolution, driven by digital technologies, is expanding and reconstructing economic and social systems worldwide (Philbeck and Davis, 2018). Indeed, a new socio-technical environment scenario characterized by global, competitive, dynamic, internationalized and digital has emerged (Kaputa et al., 2022). This opens up vast spaces for innovation in concepts, technologies, and business models across various sectors of human society. This has also brought new demands along with challenges for talent development in higher education. Digital technology profoundly influences the comprehensive transformation and development of the education system, making digital transformation an essential choice for education and a need to actively adapt to the new wave of technological revolution and societal progress (Zhong et al., 2023). Serving as key interfaces between the education system and society, higher education institutions are keys for talent development. In the current digital era, it is imperative to explore the disruptive changes brought by technological advancements to higher education from a digital transformation perspective. As the primary carriers and implementation venues of higher education, universities bear multiple responsibilities, including knowledge dissemination, talent cultivation, and scientific research. Higher education teaching activities, which encompass classroom instruction, hands-on learning, research projects implementation among intellectual communication, mostly occur within these institutions. Therefore, this paper primarily investigates how to advance the digital transformation in higher education within university teaching environments. The aims are to enhance the digital literacy and conceptual awareness of faculty and students, stimulate demand for digital services and applications, and strengthen the capacity of both educators and learners to leverage digital technologies. Ultimately, it seeks to position the digital transformation in higher education as a key driving force for the high-quality development of higher education.

Currently, there are cases in countries and higher education institutions worldwide that are actively leveraging the advantages of digital technologies such as artificial intelligence and big data to develop digital teaching tools and methods. These are towards enhancing the rationality, scientific rigor, and effectiveness of the teaching process (David et al., 2022; Thoring et al., 2018a; Bonfield et al., 2020; Tejedor et al., 2020; Martins et al., 2022). However, challenges and disruptions associated with digital transformation still persist across various elements, including teaching concepts, teaching agents, teaching models, teaching content, and teaching evaluation. These challenges necessitate adjustments to meet the developmental demands of digital transformation (García-Peñalvo, 2021). How to effectively harness digital technologies as a new driving force and steadily advance the digital transformation in higher education remains a subject worth exploring and analyzing.

Thus, this paper has been structured accordingly. First, we present an overview of existing literature on digital transformation in higher education. This article initially preset that the three most critical logics associated with the digital transformation in higher education are value logic, technological logic, and practical logic, whereas the six most critical factors related to the transformation are teaching subjects, teaching concepts, teaching models, teaching resources, teaching scenarios, and evaluation system, which need to be verified as reasonable. In the following section, we outline our methodological approach that is followed by the presentation of our case study findings and analysis. Next, based on this analysis, we propose a logical framework for the digital transformation in higher education. We then analyze potential dilemmas in this process and also identify implementation approaches of digital transformation in higher education. Finally, we present the theoretical and empirical implications of our study, outline its limitations and make suggestions/perspectives for future research.

## 2 Materials and methods

This article is an original research article with various applied methods.

### 2.1 Literature analysis and logical analysis

In terms of literature analysis, an extensive review of relevant literature was conducted. The primary sources of literature for this study were publicly accessible databases, including Google Scholar, Web of Science, Scopus, and China National Knowledge Infrastructure (CNKI). The keywords used for retrieval were digital transformation, digital transformation in education, and digital transformation in higher education. Given the substantial body of works, the authors adhered to four principles in the selection. The first priority was given to the publications of the last 7 years (2017–2024); the second is to look at journals and authors, with priority given to well-known journals and authors(in terms of impacts and reputations); the third is to look at citation rates, with priority given to those with high citation rates; and the fourth is to pay particular attention to the two types of articles that hold pro and con views on the development of the utilization of digital technology in higher education. In terms of logical analysis, the logical structure of this article comprises: an examination of three logics of digital transformation in higher education, followed by an analysis of the dilemmas related to the transformation within the context of the logical framework, an evaluation of indicators in the system of transformation implementations, and ultimately, a conclusion and recommendations.

### 2.2 Data processing and analytical procedure

To ensure a comprehensive and analytically rigorous examination of the extensive textual corpus, this study employed a tailored computational linguistics pipeline. The methodology integrated targeted web crawling with advanced computational text processing techniques. Specifically, the Qwen2.5-VL-3B-Instruct model was utilized for high-accuracy document conversion, followed by specialized lexical processing using the jieba framework. This approach enabled a systematic, data-driven identification of salient themes and conceptual patterns, forming an empirical foundation for the subsequent inductive thematic analysis that underpins the development of the triple-logic framework.

#### 2.2.1 Thematic coding and within-case analysis

Guided by the iterative approach of thematic analysis (Braun and Clarke, 2019), two primary cycles of coding were conducted:

First Cycle (Open Coding): A line-by-line analysis was performed to generate initial, descriptive codes that captured key actions and concepts.

Second Cycle (Axial Coding): The numerous initial codes were compared, sorted, and grouped into broader, more analytical categories. This process was conducted separately for each case to preserve its unique context. It was at this stage that categories pre-figured by the literature (e.g., value-oriented actions, technological investments, practical adaptations) began to emerge prominently from the data itself.

#### 2.2.2 Cross-case synthesis and framework development

The final phase involved comparing and contrasting the analytical categories across all three cases. Consistent patterns, relationships, and tensions were needed. The recurrent and interdependent presence of the three core categories—Value, Technology, and Practice—across these diverse contexts confirmed their fundamental importance. The dynamic interactions observed between them made it possible to abstract these categories into the higher-level Value Logic, Technological Logic, and Practical Logic and to model their interrelationships, thus forming the final integrated framework.

### 2.3 Case study

A case study methodology is appropriate for investigating complex, real-world phenomena in-depth and within their natural context (Crowe et al., 2011). This study employs a qualitative case study approach to examine the digital transformation process within higher education institutions. Harvard University, Tsinghua University, and the University of Melbourne were selected as main research subjects.

Three primary criteria informed the selection. First, exemplarity of digital transformation efforts: All three universities are globally recognized leaders who have publicly committed significant resources to digital transformation. Their initiatives are considered benchmark practices. Second, maximizing contextual variety: Harvard University represents a leading private, Ivy League institution within the American model. Tsinghua University represents a top-tier public university within the East Asian model, driven by both national policy and institutional ambition. The University of Melbourne represents a leading public university in a Commonwealth country, with a strong focus on internationalization and inclusive education. This diversity helps to identify common logics that persist across different systems, strengthening the framework’s general applicability. Third, data accessibility: As world-leading institutions, their strategies, reports, and outcomes are extensively documented in publicly available sources, ensuring the feasibility of conducting an in-depth, multi-faceted analysis.

## 3 A literature review on digital transformation in higher education

The digital transformation in higher education is an important component of the broader digital transformation of education (Hinings et al., 2018; Li and Song, 2022; Rampelt et al., 2019; Sepúlveda, 2020). The prerequisite for advancing the digital transformation in higher education is to maintain a scientifically informed understanding of the essence of digital transformation of education. The literature offers multiple definitions of digital transformation. A prominent perspective from EDUCAUSE frames it as a series of deep and coordinated culture, workforce, and technology shifts that enable new educational and operating models and transform an institution’s operations, strategic directions, and value proposition (Brooks and McCormack, 2020), emphasizing organizational change. Similarly, Zhu and Hu (2022b) focus on integration, defining it as the integration of digital technologies into all areas of activity, fundamentally altering the ways in which these activities are carried out by humans, and delivering value to individuals and the societies. These definitions, while useful, often lean towards either an organizational change paradigm or an element integration paradigm (Philbeck and Davis, 2018; Chen and Zhai, 2022). However, these predominant paradigms exhibit limitations. The former often overlooks the granular changes in pedagogical elements, while the latter can treat transformation as a static process of incorporation rather than a dynamic evolution.

To overcome these limitations and informed by our case analysis, this paper conceptualizes digital transformation in higher education as a process of leveraging digital technology within the higher education ecosystem to foster innovative changes in the structure, functions, and stakeholders of the system, thereby enhancing its operational vitality and elevating its service value (Zhu et al., 2022). This study thus adopts a synthesized perspective that views it as a complex, dynamic process of continuous development that capitalizes on the symbiotic coexistence and co-evolution of technological and educational ecosystems. Therefore, the digital transformation of higher education can be conceptualized through two distinct paradigms (Philbeck and Davis, 2018; Chen and Zhai, 2022; Zhu and Hu, 2022a, 2022b):

First, the organizational change paradigm. This perspective views the digital transformation in higher education as encompassing two dimensions: Digitization and transformation, regarding digital transformation as a sustainable and dynamic process of innovation. It asserts that digital transformation entails a profound alignment of people, technology, and culture, aimed at optimizing and transforming strategic direction, value propositions, and related processes.

Second, the element integration paradigm. This viewpoint regards digital transformation in higher education as a static process, emphasizing the incorporation of digital technologies into teaching, learning, assessment, and organizational structures, thereby precipitating educational reform (Chen and Zhai, 2022).

These two distinct paradigms diverge in their perspectives on whether the digital transformation in higher education constitutes a dynamic process, the critical factors of transformation, and the role digital technology plays within this process, with each viewpoint exhibiting relative limitations. Therefore, by synthesizing the strengths of both approaches, this paper contends that the digital transformation of higher education is neither a simplistic sequential addition of digitization followed by transformation, nor a static process of integration. Rather, it refers to a process of leveraging the advantages of digital technology within the higher education ecosystem to foster innovative changes in the structure, functions, and stakeholders of the higher education system, thereby enhancing its operational vitality and elevating its service value (Zhu et al., 2022).

Drawing upon the perspectives of previous scholars, this paper posits that the digital transformation in higher education represents a new paradigm that reconstructs higher education based on the concept of digital transformation. It is characterized as a complex, dynamic process of continuous development, utilizing digital technologies to reform higher education. This process involves integrating digital technologies into various domains and levels of teaching within higher education institutions, with a focus on enhancing the digital literacy of faculty and students, transforming teaching concepts, optimizing teaching models, updating teaching resources, and reforming teaching evaluation systems. This transformation fully capitalizes on the strengths of digital technology to provide the higher education system with greater operational vitality and elevated service value. It does not only underscore the deep integration of digital technologies into higher education but also highlights the symbiotic coexistence and co-evolution of technological ecosystems and educational ecosystems.

## 4 Case study: finding the main components of the logical framework of digital transformation in higher education

The digital transformation in higher education represents a holistic, systematic, and structural shift grounded in the practical realities of teaching and learning within higher education institutions. It does not entail a comprehensive deconstruction or outright rejection of traditional higher education but it rather emphasizes uncovering the latent interconnections among various elements in the process.

### 4.1 Digital literacy enhancement at Harvard University

Harvard University has prioritized the enhancement of digital literacy among both faculty and students as part of its broader educational transformation. This aligns with the fundamental value pursuit in higher education’s digital transformation, which emphasizes the cultivation of digital citizens. Harvard University Learning Technologies and Innovation department offers training and resources to help faculty integrate digital tools into their teaching. Additionally, Harvard provides numerous (University, H., 2024) online courses (through platforms like edX) that promote digital literacy among its students and the global learning community. CS50 (Liu et al., 2025), Harvard’s popular introductory computer science course, incorporates real-time coding feedback through AI-powered tools, fostering the development of digital skills (e.g., data literacy, coding, problem-solving, and continuous learning). The core of Harvard’s approach revolves around the value of enhancing digital literacy, making it a priority in their educational reforms. This aligns with the global need to equip students and faculty (University, H., 2023) with the necessary ability to thrive in a digital society. In addition, Harvard’s technological adoption (AI and cloud-based learning tools) supports these values by providing scalable solutions for skill development. In advance, the interactive feedback mechanism/tools of Harvard’s online courses helps refine and adapt digital literacy programs based on the reality of student needs and performance.

### 4.2 Digital transformation at Tsinghua University

Tsinghua University in China has been at the forefront of integrating digital technology in its curriculum, with a focus on enhancing digital literacy and removing barriers to technology adoption. Tsinghua has implemented a Smart Campus system, using Rain Classroom, which integrates AI-based learning analytics and personalized feedback to enhance the digital skills of both faculty and students. The university runs digital literacy workshops, teaching both staff and students not only how to use technology but also how to ethically engage in the digital world, preparing them for future roles as digital citizens. (Institute of Education and UNESCO, 2022) Tsinghua University digital transformation process involves a concerted effort to eliminate barriers like outdated teaching resources and resistance from educators unfamiliar with new technologies. Tsinghua University prioritizes the digital literacy of faculty and students as part of its larger educational mission, ensuring that everyone involved in the teaching process has the necessary digital ability. The university employs state-of-the-art technologies such as AI and IoT to create a seamless learning environment. Feedback loops, in the form of evaluations from both students and faculty, enable a continuous improvement of the university’s digital tools and strategies.

### 4.3 The University of Melbourne—overcoming barriers towards digital transformation

The University of Melbourne (Mark et al., 2022) in Australia is addressing barriers to digital transformation by establishing supportive infrastructures and addressing the resistance to change among faculty members. The University launched the Digital Learning Strategy with a focus on enhancing the capacity of staff through professional development programs that help them incorporate digital tools into their teaching practices. They have also implemented a Digital Education Infrastructure, which includes an extensive online learning platform and digital resources that empower students and faculty to adapt to new learning environments. To address normative barriers, the university has undertaken a comprehensive assessment of existing technological tools, pedagogy, and resources, towards making these elements more accessible and equitable. The university’s primary focus on breaking down barriers to digital adoption ensures that its teaching and learning process is more inclusive and adaptable to the digital age. The emphasis on scalable technological solutions supports both faculty development and student engagement. The university’s interactive approach, including continuous feedback from stakeholders and following the testing of new digital solutions, allows for practical adjustments and refinements.

From the illustrative cases above, we found that the digital transformation in higher education requires a structured yet flexible approach. A triple logic framework—anchored in value, technology, and practice—provides a robust model for sustainable and impactful digital transformation. Their applicability and effectiveness are illustrated in cases at Harvard University, Tsinghua University and at the University of Melbourne.

## 5 A logical framework of digital transformation in higher education

Value principles, technological intermediaries, and practical methods are identified as three pivotal components, requiring mutual reinforcement and coordinated alignment throughout the transformation process. Existing frameworks for digital transformation in higher education often focus on either the technological (Scherer et al., 2019; Park et al., 2022) or organizational aspects (Law et al., 2018; Vuorikari et al., 2022). Whereas these models provide valuable insights, they fail to capture the holistic and interactive nature of digital transformation in higher education.

**Figure 1:**
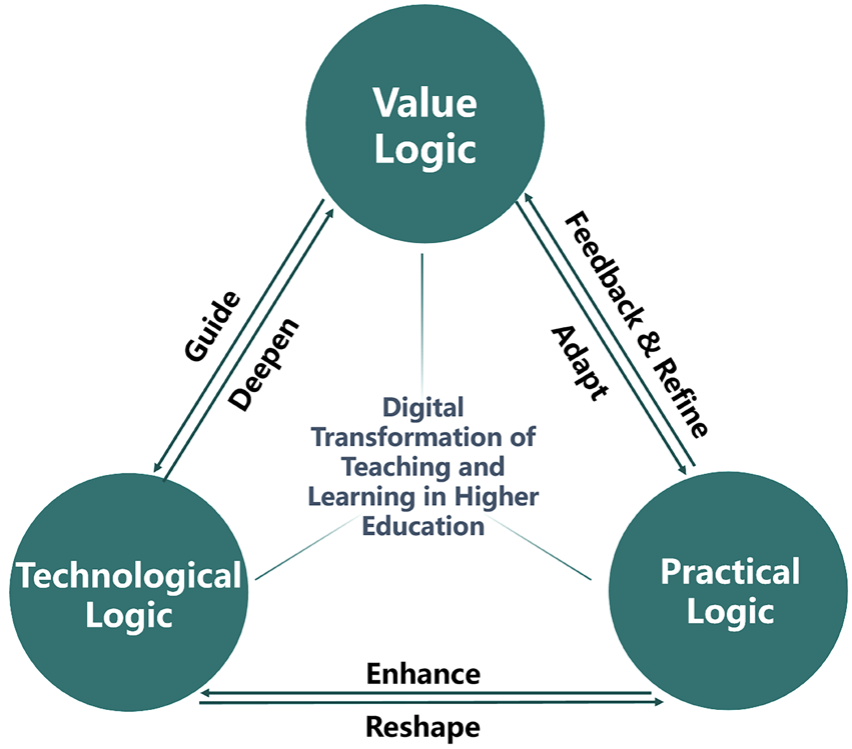
Three logics of digital transformation in higher education.

Thus, in this article a sustainable and integrated framework for the digital transformation in higher education is also proposed, comprising the value logic, technological logic, and practical logic. The triple logic framework overcomes these limitations by integrating value-driven, technology-oriented, and practice-based perspectives. Within this framework, value logic guides technological logic and is further refined during technological application. Technological logic strengthens practical logic and reshapes it through teaching practices, while practical logic evolves according to value logic and provides ongoing feedback to enhance and refine value logic based on experiential insights (see Figure 1). This framework seeks to transform the empowering effects of digital technology into new momentum for advancing the high-quality development of higher education.

### 5.1 The value logic

The value logic refers to the value pursuits held by society, higher education institutions, and their faculty and students, as well as the value norms that should be adhered to during the process of digital transformation in higher education. These elements collectively play a guiding role in the digital transformation of higher education systems, serving as the subjective driving force behind its advancement (Wu, 2024).

Positioning the comprehensive enhancement of digital literacy among university teachers and students as the core support of transformation constitutes the fundamental value pursuit of higher education digital transformation. The Value is not a property of the object itself but is primarily assigned or imputed to the object by the judgment of the subject in the value relationship between them. Indeed, an object has no positive or negative value, nor does its nature and measures, unless a subject bestows value on it through judgment (Engineering). Teaching and learning, as a cohesive process involving both faculty and students to achieve educational objectives, represent an organic whole. Faculty and students, as the primary agents of teaching and learning, are critical elements in the transformation process. The wave of digital transformation has driven the reform of traditional higher education teaching and learning, with the cultivation of digital citizens possessing adequate digital literacy becoming a new educational orientation. Digital literacy encompasses fundamental knowledge and skills related to digital technology, information and data literacy, the ability to communicate and collaborate using digital technology, the ability to create digital content, awareness of digital security and digital ethics, the capacity for continuous learning, problem-solving, reflection, and self-improvement through digital technology, as well as specialized digital knowledge and ability (Commission, 2018; Law et al., 2018). Throughout the digital transformation process, teaching agents actively engage with other components—such as teaching objectives, content, and activities—forming interconnections and mutual influences. Therefore, the primary value pursuit of higher education’s digital transformation must prioritize the elevation of teaching agents’ digital literacy, specifically that of university faculty and students, as the central support of the transformation. Moreover, it is essential to fully understand the intrinsic connections and interactions among various elements of the teaching process, thereby optimizing the traditional teaching structure. This value pursuit not only acknowledges the instrumental value of digital technology for individuals and society but also adheres to its inherent value in enabling collective human intelligence through digital empowerment. Thus, the value pursuit of digital transformation in higher education transcends mere technological and data-oriented changes. It aspires to enhance the core ability of teaching agents in universities, providing them a strong ability to adapt to new tools and excel in the digital era.

Addressing the objective barriers that constrain the digital transformation in higher education should be established as a core normative value of this transformation. Normative values exert a regulatory influence on the behaviors of organizations and individuals, intrinsically guiding the orderly development and evolution of organizations towards a specific direction. Establishing the removal of barriers to digital transformation as a normative value underscores that digital transformation is not merely a process of technological innovation or pedagogical reform but also a profound reassessment with a reshape of the underlying values of higher education.

The digital transformation in higher education entails multidimensional changes, encompassing not only the digital literacy of university faculty and students as key teaching agents but also objective aspects such as teaching models, resources, and evaluation systems. Failure to address the barriers impeding transformation risks stagnation or counterproductive outcomes. Therefore, positioning the resolution of these constraints as a normative value is a critical condition for ensuring the genuine realization of digital transformation in higher education.

### 5.2 The technological logic

Technological logic refers to the fundamental principles, modes of thinking, and operational mechanisms adhered to during the application of technology. It is typically centered on improving efficiency, optimizing processes, or enhancing functionality. It emphasizes the inherent capabilities and characteristics of technology, prioritizing the ways in which it can be utilized and applied to achieve specific goals, often focusing on functionality, feasibility, and efficiency. Technological factors represent the core distinction between teaching-and-learning in digital transformation and traditional educational methods. As Marx asserted, technology arises and evolves within practical activities aimed at meeting human needs. Essentially, technology is a human creation—a tool and means for sustaining and advancing human existence and development (Liu, 2017). Thus, technology inherently possesses a teleological character, as it is invariably guided by the needs of human development and the resolution of practical problems. This teleological nature reflects the social responsibility imbued in technology by humanity (Wang and Cai, 2024). This perspective aligns seamlessly with the value logic of enhancing the digital literacy of faculty and students and addressing the objective barriers to the digital transformation in higher education. Accordingly, the technological logic underpinning the digital transformation in higher education, guided by value logic, originates from the developmental needs of students and faculty and explores the relationship between higher education and technology. At its core, this relationship seeks to address how to properly balance technology with the principles of teaching during digital transformation, while safeguarding the rational boundaries between technology and humanity. Therefore, the technological logic of digital transformation in higher education, guided by value logic, involves an exploration of the relationship between higher education and technology, rooted in the developmental needs of faculty and students. At the core of this relationship lies the critical task of appropriately managing the interplay between technology and the principles of pedagogy during the process of digital transformation while steadfastly maintaining the rational boundaries between technology and humanity.

Preventing the superficial integration of digital technology with higher education is essential. Over-reliance on existing digital technologies risks leading education into the trap of technological misuse, where the educational process is reduced to mere data and statistical analysis, overlooking the complexity and diversity of students’ intellectual development. Such an approach may stagnate innovation in higher education, rendering it incapable of meeting evolving societal needs and the diverse developmental requirements of students. Additionally, when educators regard technology as the sole solution, they may neglect the creative potential of teachers as well as the innovation of educational content, resulting in rigid and formulaic teaching methods. This often manifests as an overemphasis on low-level uses of digital technology rather than a genuine and a student-centered transformation. The term superficial integration refers to the mere patchwork application of technological tools in the educational process, leading to fragmented and hollow educational experiences that fail to achieve their intended outcomes. The influence of digital technology on higher education often begins with the contextual application of technology. For instance, during the pandemic, higher education institutions employed cloud platforms, the Internet of Things, big data, blockchain, and other modern information technologies to support online teaching effectiveness. However, in many cases, teaching interactions between faculty and students passively conformed to digital applications such as big data analysis, virtual reality, and online classrooms without deeper integration. What was overlooked in this process were the fundamental changes brought about by the embedding of digital technologies into the teaching domain, including transformations in teaching relationships, environments, contents, models, and evaluations. Avoiding over-reliance on digital technology and preventing its superficial integration with higher education are essential for preserving the essence of education, sustaining innovation, achieving educational equity, and fostering students’ holistic development. Digital technology should serve as a powerful tool in higher education, not as a dominating force. It should support the achievement of educational objectives rather than supplanting the essence of education itself. Higher education needs to integrate technology thoughtfully to enhance educational outcomes and learning experiences, rather than allowing technology to constrain the educational process. Thus, during the digital transformation in higher education, it is imperative to avoid the superficial integration of digital technology with teaching practices.

Emphasizing the balance between the instrumental rationality of digital technology and the ecosystem of higher education. Another problem solved by technological logic is how to reconcile the inherent tension between the instrumental rationality of digital tools in the digital transformation process and the human nature of the digital practice of higher education subjects in the process of digital transformation. As a subsystem of the broader societal system, higher education serves as a critical platform for talent cultivation and cultural transmission, providing the human and cultural foundations for societal functioning. Consequently, its digital transformation must center on the core participants in education: Teachers and students. Guided by value logic, digital technology in higher education focuses on shaping the core ability of faculty and students, enabling them to become future digital citizens equipped with essential abilities and qualities. At the societal level, digital technologies in the context of higher education’s digital transformation address the demands of the digital era-related productivity, thereby reinforcing their societal value. At the individual level, these technologies prioritize the personalized learning and development needs of students and the enhancement of teachers’ digital ability (Tay and Low, 2017). This shift transforms the role of technology from being supply-driven to demand-driven, fostering a mutually reinforcing relationship between the technological ecosystem and the higher education ecosystem. This complementary dynamic facilitates the symbiotic integration of digital technologies and educational practices, rooted in the practical essence of digital education. It enables the instrumental rationality of digital tools to work in tandem with the ecological logic of higher education, maximizing their synergistic potential in the construction of a robust higher education ecosystem. Consequently, the instrumental rationality of technology is harnessed to complement and enhance the developmental needs of higher education, ensuring a harmonious and dynamic interplay between technological and educational ecosystems.

### 5.3 The practical logic

The concept of practical logic originates from the habitus theory proposed by the French sociologist Pierre Bourdieu (Bourdieu, 1990). Bourdieu posited that habitus refers to the patterns of thought and behaviour formed through prolonged socialization processes, which are manifested in practice. Practical logic, therefore, represents the realization of habitus in specific practices, embodying the behavioural patterns and response mechanisms of individuals in concrete situations. The practical logic refers to the set of behavioural principles employed by social actors in specific contexts, shaped by their cultural background, values, beliefs, and habitual practices. This logic influences how individuals interpret the world, take actions, and interact with others. It is not merely a way of thinking but also encompasses specific actions and behaviours within daily life.

As highlighted in the discussion of technological logic, the fundamental changes in teaching relationships, teaching environments, content, methods, and evaluation introduced by the integration of digital technology into higher education are often overlooked by current institutions. This oversight frequently results in a disconnect between practice and educational philosophy. Consequently, the challenge of balancing the instrumental rationality of digital technology with the ecological dynamics of higher education, as emphasized by technical logic, places heightened demands on the practical value of digital transformation in higher education. In this transformation, faculty and students must adjust their practical logic in alignment with value logic, shifting from conceptual changes to concrete measures that fully meet the demands of the digital era in higher education. During this process, the generation, dissemination, and application of knowledge within higher education exhibit significant openness and diversity. Traditional teacher-centred models of knowledge delivery are increasingly giving way to diversified and multifaceted sources of knowledge acquisition. Digital technology, with its multidimensional capabilities, enables new possibilities in educational practices. Therefore, this paper posits that leveraging the multidimensionality of digital technology to deconstruct the singularity of the current higher education represents the practical logic of its digital transformation.

The practical endeavours associated with the digital transformation of teaching and learning in higher education do not follow a straightforward and linear progression. Under the impact of digital technologies, the internal structural elements exhibit increasingly complex and diverse tendencies. Consequently, deconstructing two forms of singularity within higher education practices emerges as a key aspect of the practical logic of digital transformation: first, transitioning from a teacher-dominated teaching model; second, reforming the unidimensional and narrow approach to educational evaluation. On the one hand, digital technology serves as a means for practicing subjects to understand and participate in the reform of higher education teaching and learning, and its participants include two main subjects, teachers and students within a traditional teaching practice. In this practice, teachers are the main subjects of teaching and students are the main subjects of learning. However, in the digital era, students have assumed new roles, enabled by digital technology to diversify learning processes and feedback mechanisms (Jackson, 2019). In the practice of higher education, this shift from a teacher-centred model to a dual-subject model involving both teachers and students enhances the digital capabilities of both groups, equipping them to address the dynamic and multifaceted challenges of the digital age. Furthermore, digital technology has broken the temporal and spatial constraints of higher education, fostering a new paradigm of ubiquitous learning that allows learning anytime, anywhere, and for anyone. This development significantly enriches the content of education, providing learners with a vast array of resources to explore and select. Consequently, standardized and uniform teaching models should evolve accordingly.

On the other hand, digital technology introduces disruptive innovation and multidimensional restructuring to higher education evaluation systems (Eliveria et al., 2019). Traditional evaluation methods are often overly simplistic and limited, failing to comprehensively capture students’ learning outcomes and teachers’ instructional effectiveness. Higher education evaluation must transition toward an intelligent, diversified evaluation mechanism, focusing on both teacher and student assessment. This transformation involves exploring multi-stakeholder participation and stratified evaluation standards, offering a pluralistic perspective that continuously informs the practical approach to digital transformation. By adopting the logic of addressing singularity with digital technology and deconstructing singularity through diversified practices, higher education can, in practice, achieve genuine personalization and adaptability in teaching and learning.

### 5.4 The relationship between the three logics

As visualized in Figure 1, the three logics constitutes a dynamic, tripartite framework elucidating the digital transformation of teaching and learning in higher education. This framework posits that transformation is driven by the continuous, synergistic interaction between value logic, technological logic, and practical logic. Value logic, embodying the core why or normative principles, serves to guide the entire process by establishing strategic direction and pedagogical goals. Technological logic, representing the how, is both guided by these values and, in turn, works to deepen them by revealing new possibilities and constraints through its application. Simultaneously, technological logic enhances and reshapes practical logic—the what of everyday teaching practices—by providing new tools and methods. Finally, practical logic is not a passive endpoint; it continuously evolves according to value logic while also generating essential feedback from real-world implementation. This feedback refines and adapts the overarching value logic, ensuring it remains relevant and effective. Thus, the model depicts a sustainable, iterative cycle where values, technology, and practice are co-constitutive, moving beyond a simplistic, techno-centric view to offer a holistic lens for understanding and orchestrating digital transformation.

## 6 Practical dilemmas

Guided by the triple-logic framework, our analysis of the case studies reveals that the current digital transformation in higher education is fraught with multifaceted dilemmas. These challenges are not isolated but are interconnected manifestations of misalignments between value aspirations, technological implementations, and practical adaptations. The aforementioned value, technological, and practical logics collectively place specific demands on the digital literacy of faculty and students, teaching concepts, applications of digital technology in instructional settings, teaching models, and feedback mechanisms within the real-world educational environment. From the perspectives of this framework, the current challenges are primarily reflected in the following aspects.

### 6.1 Disparate digital literacy of teachers and student and accessibility of teaching resources

Mariya Gabriel, EU Commissioner for The Digital Economy and Society has pointed out that 90% of future jobs require some level of digital literacy (Commission, 2018). Digital ability (Anusca, 2013) has also been recognized by the European Union as one of the eight key competences for lifelong learning. Therefore, students in higher education institutions, as future key contributors to the workforce, must continually enhance their digital literacy through learning practices during their academic tenure. Teachers in higher education institutions, as guides and facilitators, should also improve their digital ability to meet the demands of a digital age society for talent development. However, globally, several common challenges are encountered, including technological barriers (related to institutional ICT resources and capabilities), personal barriers (related to proficiency with technology), and difficulties in teaching and learning (such as promoting student engagement and collaborative efforts), including issues like low student participation rates (Qureshi et al., 2020).

The dilemma of digital literacy among teachers lies in the insufficient digital support available to faculty in higher education institutions. The digital transformation in higher education relies on a teaching workforce equipped with digital capabilities and skills. However, as digital education concepts remain in an exploratory stage, the lack of digital talent within the faculty pool fails to meet the demands of transformation. A survey conducted by Mhlanga D. et al. revealed that two-thirds of higher education institutions reported Teachers face challenges regarding the uncertain availability of digital infrastructure, personal digital ability, and digital teaching and learning processes (Kanyane, 2023). Moreover, due to insufficient training on the use of digital resources, many teachers are unfamiliar with digital teaching platforms and struggle to fully utilize available digital tools, leading to a lack of confidence in teaching within digital environments (Guillén-Gámez et al., 2021). Additionally, consensus has yet to be reached on the core elements of frameworks for evaluating teachers’ digital ability, and existing frameworks are not fully aligned with the practical teaching contexts in certain regions (Devisakti et al., 2024). As a result, university teachers are unable to effectively use these frameworks for self-assessment and feedback.

The dilemma of digital literacy among students is primarily manifested via two aspects. On the one hand, disparities in personal learning interests and paces, combined with economic differences among regions, have resulted in a digital divide between schools and among students (Xiao, 2019). On the other hand, the application of generative artificial intelligence and other digital technologies in the learning process has partially replaced students’ cognitive and physical efforts, leading directly to the presentation of final results. Over time, students may develop excessive reliance on generative AI due to its speed, rich content, and interactive capabilities. This overdependence risks positioning digital technology as a central player in the learning process, even fostering the misconception among students that digital tools can replace teachers. Such trends exacerbate the focus on knowledge transmission at the expense of the fundamental purpose of higher education—to cultivate well-rounded individuals. Consequently, the risks of hindering students’ emotional development and the formation of healthy, balanced personalities are significantly increased.

### 6.2 Conceptual disagreements and misunderstandings of teaching and learning

A second critical dilemma lies in the realm of value logic, where a profound disconnect exists between the transformative potential of digitalization and the conceptual understandings that guide daily practice. This misalignment manifests as three interrelated challenges:

Ambiguous Strategic Positioning: Our analysis identifies a significant gap between the official leading role of digital transformation and its perception as merely a supporting or assisting tool in the cognition and teaching practices of many stakeholders. This ambiguity, as noted in the literature (Engineering, 2020; Sjöberg and Lilja, 2019; Link et al., 2020; Dar, 2022; Alenezi et al., 2023; Johinke et al., 2023), stems from varying levels of awareness among leaders and staff across different subunits of large institutions, preventing a unified commitment to the core value proposition of transformation.

The Pitfall of Techno centrism: The observed phenomenon of digital transformation for the sake of digital transformation exemplifies a distortion of technological logic, where an excessive reliance on technology overshadows the value-driven goals of student development. This aligns with García-Peñalvo’s (2021) caution against the dark side of digital transformation, where a focus on hardware over content and technology over students leads to passive learning behaviors and neglects long-term impacts.

Teacher Identity Alienation and Ambivalence: The shifting roles and declining professional authority resulting from digital transformation have intensified teacher ambivalence (Sjöberg and Lilja, 2019), leading to confusion and a conservative attitude toward change. This identity alienation (Law et al., 2018; Link et al., 2020; Dar, 2022) represents a critical failure to align the practical logic of teaching with the new value expectations of the digital era, hindering the adoption of new methods needed to meet diverse student needs.

### 6.3 Homogeneous teaching scenarios and models

Currently, the higher education system remains constrained by established inertia, with the predominant teaching models in universities largely adhering to a teacher-student binary structure rather than transitioning to a teacher-technology-student triadic one. This limitation reflects the ongoing challenges posed by the singular physical form of universities and the prevailing homogeneous teaching model, which hinders the development of differentiated instruction. A study examined how the role of digital transformation is framed in the strategic development plans of 75 top universities in China (Xiao, 2019) found which can be seen in HEI of other countries that there is not enough incentive to use digital technologies to serve a wider community and to build technology-enhanced research capacity. There is also limited evidence of open, flexible, distributed, and disaggregated learning encouraged.

From the perspective of institutional form, the physical boundaries between higher education institutions are expected to dissolve with the advent of digital transformation. Teaching scenarios should transcend the constraints of physical spaces, evolving toward diversified hybrid models that seamlessly integrate online and offline environments. Regarding teaching models, interconnectivity among institutions and between universities and society should facilitate resource sharing across faculty, courses, facilities, and services. This interconnectedness fosters the diversification of learning environments and addresses the increasing demand for personalized learning, enabling precise and tailored instruction based on individual student needs.

The process of higher education digital transformation is, in essence, a process of empowering learners with greater control over their education. By reconstructing teaching models, a personalized talent cultivation paradigm can be achieved. However, deeply entrenched traditional practices continue to persist. For example, technological determinism has long dominated educational perspectives, while the lack of corresponding capacity-building and updated management systems has hindered the effective application of digital technologies. Consequently, the conventional structure of physical classrooms and uniform curricula—serving dozens to hundreds of students across entire classes or programs—remains firmly in place. As a result, higher education has yet to leverage emerging digital technologies to construct truly cohesive and unified new teaching scenarios and models. The temporal and spatial boundaries of higher education institutions remain unbroken, making it difficult to achieve differentiated and personalized learning.

### 6.4 Lack of a scientific and mature teaching and learning evaluation system in higher education

The digital transformation in higher education is a dynamic and continuously evolving systemic process that necessitates the role of evaluation in providing ongoing feedback and adjustment. It is essential to monitor and assess the development of digital ability among faculty and students in real-time, diagnosing and correcting adverse factors in teaching practices promptly. The feedback mechanism aims to inform and guide the theory and the practice during the transformation process. While numerous higher education institutions and educational organizations worldwide have made significant progress in transformation policies and educational infrastructure, the effectiveness of changes in teaching methods remains less evident. One of the key reasons can be found to be the lack of a scientifically robust evaluation system that aligns with the goals and value orientations of digital education. The current evaluation systems lack clarity in terms of evaluative agents, indicators, and methods, making it difficult to assess the maturity of the digital transformation in higher education teaching (Zhong et al., 2023).

## 7 Implementation approaches of digital transformation in higher education

**Figure 2:**
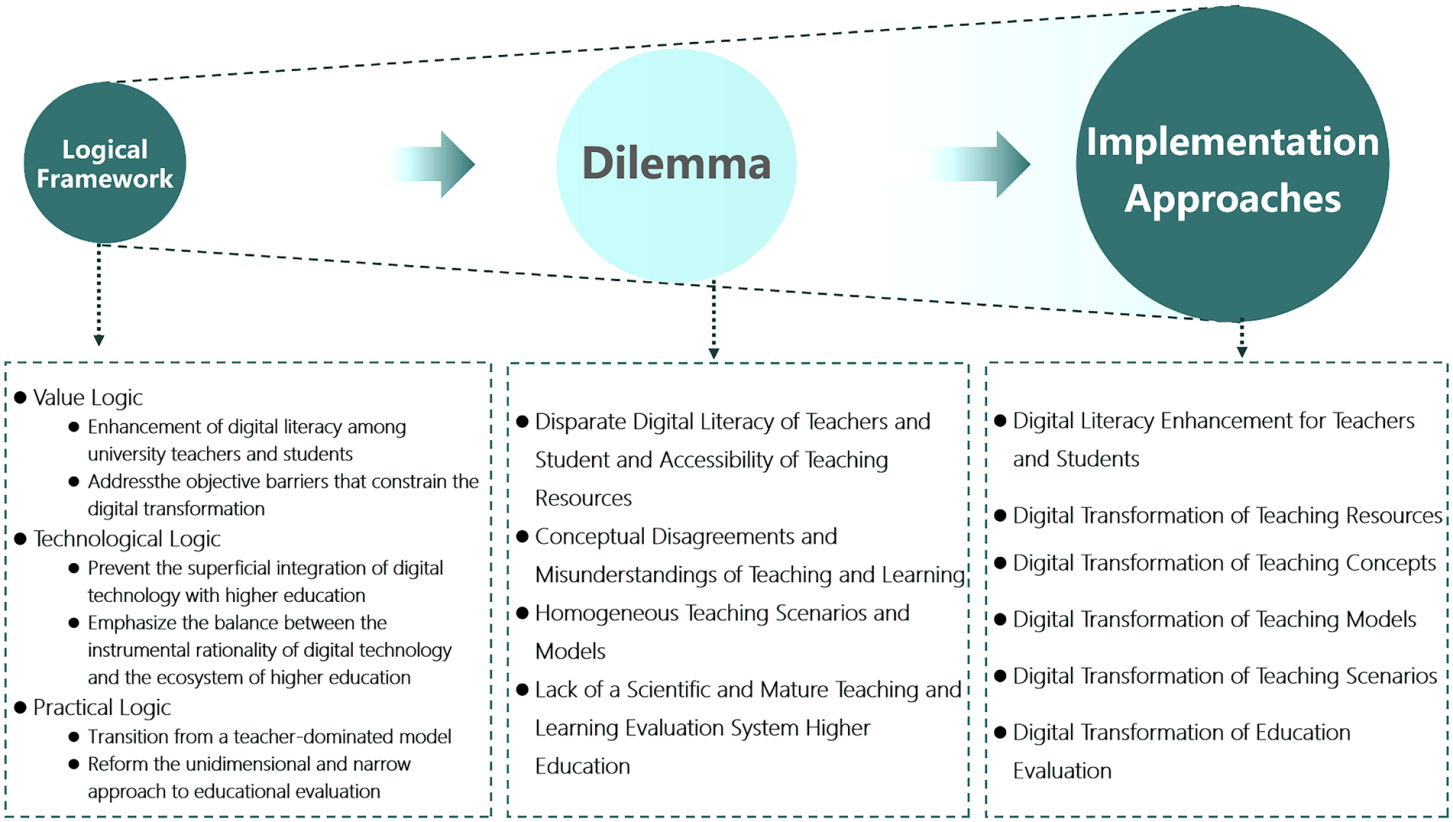
From logical framework to solving dilemmas via implementation strategies.

As previously outlined in the logical framework, during the transformation process, teaching subjects actively establish connections and mutual influences with other elements, including teaching objectives, content, and activities. The digital transformation in higher education represents a systemic endeavor that necessitates comprehensive advancements across the dimensions of value, technology, and practice. All components of education—such as teaching subjects, content, methods, and evaluation of educational outcomes—must undergo a swift transition from the relatively stable traditional models shaped by the industrial society to new scenarios and environments constructed by digital technologies and their applications. This transformative process entails the reconfiguration of teaching concepts, environments, and teacher-student relationships, alongside comprehensive reforms in teaching models and evaluation mechanisms (as illustrated in Figure 2).

Beyond simply adopting advanced digital technologies, digital transformation involves revolutionary and holistic shifts within the organization—its core lies in fully leveraging the changes and opportunities brought by the combination of digital technologies and their overall influence on society, and promoting such changes in a strategic and prioritized manner based on current and future needs (Gkrimpizi et al., 2023). Regarding implementation approaches, this article references research findings and practices from multiple countries and regions (Ministry of Education, 2010; Thoring et al., 2018a, 2018b; Wildan Zulfikar et al., 2018; Bai and Shen, 2019; Blake, 2019; König et al., 2020; UNESCO Office Bangkok and Regional Bureau for Education in Asia and the Pacific and International Centre for Higher Education Innovation, 2021; Education, 2022; Wang et al., 2022), including those from UNESCO (UNESCO, 2020; UNESCO Office Bangkok and Regional Bureau for Education in Asia and the Pacific and International Centre for Higher Education Innovation, 2021; Nations, U, 2022; Vuorikari et al., 2022), the European Union (Anusca, 2013; Qureshi et al., 2020; Commission, 2021; Comission, 2022; Farias-Gaytan et al., 2022; Vuorikari et al., 2022; Nations, 2023), and various higher education institutions (Ellis-Chadwick and Chaffey, 2012; Chan et al., 2017; Azarenko et al., 2018; Benavides et al., 2020; Berguerand, 2020; Farias-Gaytan et al., 2022; Li et al., 2022; Bisri et al., 2023; Farias-Gaytan et al., 2023) and finally synthesizes the following transitional paths as a whole.

**Figure 3:**
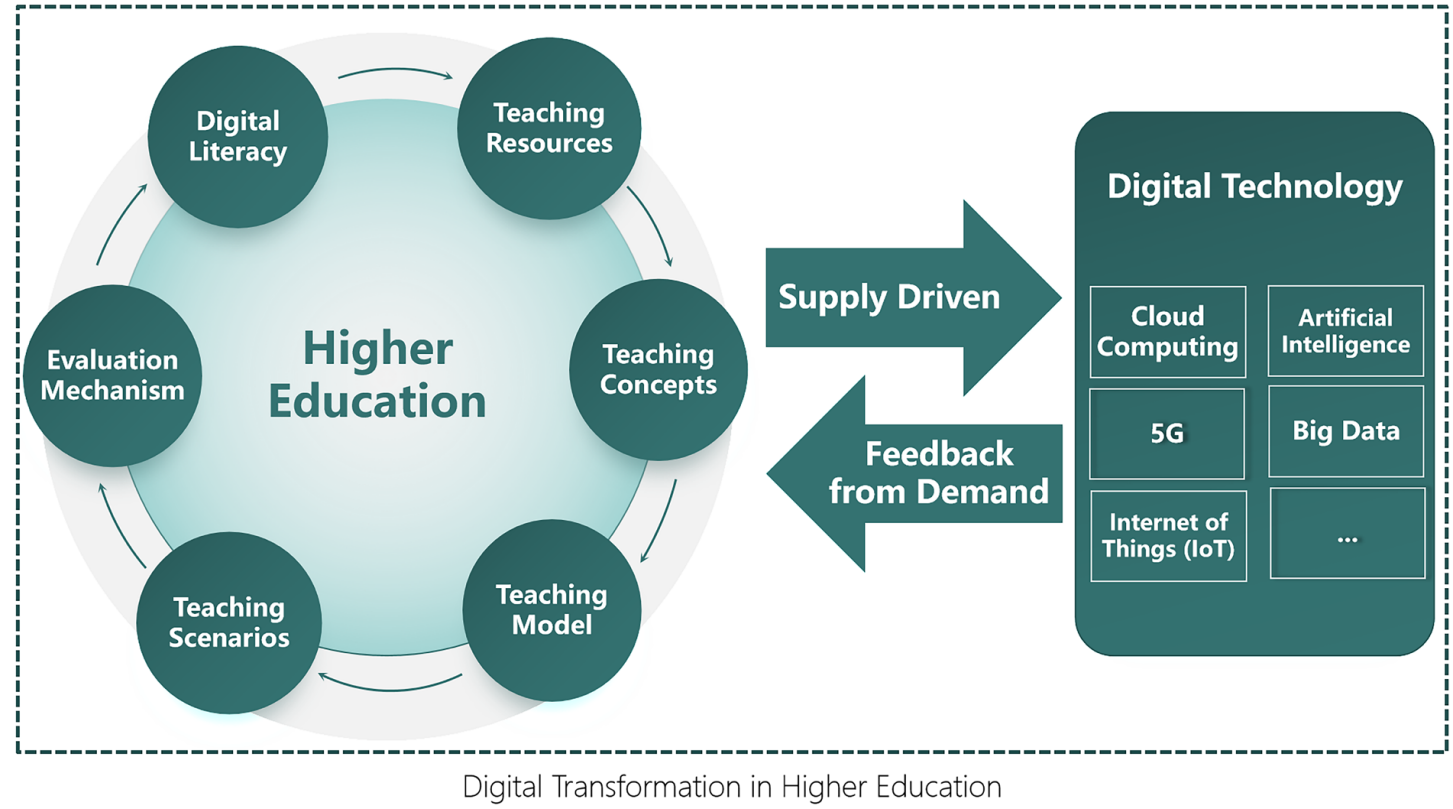
The interaction between core teaching dimensions and digital technology.

As visualized in Figure 3, the process is both supply-driven by the rapid advancement of foundational digital technologies and feedback-driven by the evolving demands of the educational ecosystem. The six implementation pathways are categorized into three core domains. Foundational Elements (The “What”): This includes the transformation of Teaching Resources and the enhancement of Digital Literacy for all stakeholders, which form the essential base for any digital initiative. Process and Practice (The “How”): This encompasses the evolution of Teaching Concepts, Teaching Models, and Teaching Scenarios, representing the practical application of digital tools in pedagogical design and delivery. Evaluation and Improvement (The “How Well”): The Evaluation Mechanism closes the loop, providing critical feedback to assess efficacy and inform continuous improvement across all other components. Ultimately, the synergistic interaction of all these pathways—fueled by technology and guided by pedagogical value—converges to achieve the overarching goal: a comprehensive and sustainable Digital Transformation in Higher Education.

### 7.1 Digital literacy enhancement for teachers and students

Our case analysis underscores that enhancing digital literacy is the foremost implementation approach, a finding that aligns with global priorities. Specifically, we propose that promoting the active participation of teachers requires three key actions:

Implementing continuous, practice-oriented learning programs. Unlike traditional training, these programs must focus on building the capacity to integrate digital technology seamlessly with disciplinary knowledge, encompassing areas from digital resource development to data-driven evaluation. This approach finds support in the work of Cárdenas-Robledo and Peña-Ayala (2018), who stress the importance of ongoing pedagogical support.

Leveraging digital technologies to create optimized professional spaces. We suggest establishing a digital platform for faculty development that enables teachers to collect, analyze, and evaluate instructional data. This supports the formation of a comprehensive feedback loop for professional growth, a concept exemplified by the practice of establishing professional e-portfolios.

Cultivating digital security awareness and respect for digital ethics among higher education students should be strengthened. Students should be educated or trained in digital communication and digital governance to create a virtuous new digital education ecosystem.

### 7.2 Digital transformation of teaching resources

The digital transformation of teaching resources in higher education primarily encompasses two aspects.

First, the transformation of teaching resource carriers. This involves converting physical, paper-based teaching materials into digital formats that can be accessed through digital terminals, dynamically updated, and capable of recording interactive traces in real-time. This transformation goes beyond merely uploading teaching slides or converting book chapters into online modules. Instead, the content structure should exhibit dynamic, open, and unstructured characteristics to accommodate diverse learning needs (Bygstad et al., 2022). Learning resources serve as a vital foundation for teaching activities, and their digitization must extend beyond simple backups of teaching videos or documents on digital platforms. It is essential to establish connections among resources, define utilization pathways, and support personalized learner engagement. This approach ensures that learners do more than just browse textual content or watch instructional videos; it enables the formation of interactive communities between educators and learners. Furthermore, data collection and analysis of the learning process should be implemented to allow educators to monitor learners’ progress and provide tailored support in real-time.

Second, the digitization of teaching resource content addresses the multifaceted nature of higher education, characterized by a wide variety of academic disciplines, diverse instructional elements, and multiple specifications for talent development. Advancing comprehensive digital transformation with teaching digitization at its core requires not only the digitization of resource carriers but also the synchronized digitization of content such as experimental data, teaching research materials, and library collections. These comprehensive digital repositories are essential for supporting the broader digital ecosystem of higher education. Such integration facilitates an all-encompassing and multi-dimensional transformation of teaching practices, thereby enabling a full-scale digital transformation within the higher education systems.

### 7.3 Digital transformation of teaching concepts

The digital transformation involves not only technological changes but also a revolution in thinking philosophy and concepts. The current era is one of co-evolution between humans and digital technology. It is essential to establish the idea of integrating digital technology into education. It must be fully recognized that the core of value innovation in educational digital transformation lies not in technological innovation perse, but in the development of human capabilities and the cultivation of thinking. A digital concept of teaching and learning in higher education that aligns with the needs of a digital society must be established.

First, a transformation in the perception of digital identity among teachers in higher education institutions is required. Higher education teachers must embrace a transformative shift in their professional identity, moving from the traditional role of teacher to the broader and more dynamic role of educator (CoSN, 2023). This conceptual evolution emphasizes that, in the era of digital transformation, teachers are no longer mere transmitters of knowledge. Instead, they are composite educators who embody multiple roles, including learner, collaborator, guide, and facilitator. This transition necessitates both digital teaching ability and higher-order digital creativity, requiring teachers to advance from passively utilizing digital tools to actively creating and innovating within digital education spaces.

Second, the encouragement of self-directed learning and personalization for learners at higher education level is another key point. It is achieved with higher education teachers embracing a transformative shift in their professional identity from the traditional role of teacher to the broader and more dynamic role of educator, in parallel, the role of students is also redefined. Teachers are no longer mere knowledge dispensers, and students are no longer passive recipients of information. As highlighted in the Consortium for School Networking’s Driving K–12 Innovation Report (2024): Challenges, Trends, and Technologies (Cavanagh et al., 2020) a critical trend and catalyst for accelerating innovation is learner agency, where students actively take ownership of their learning process. This redefinition shifts the role of student to learner, reflecting a proactive and empowered approach to education. Digital technology facilitates self-directed learning by providing accessible resources and diverse learning modalities, transcending the limitations of traditional physical classrooms. Within this environment, learners can transition from passive participants to active innovators. Encouraging learners to start with their digital literacy needs, digital tools and data can be leveraged to establish learning trajectories, autonomously track progress, and develop personalized learning plans with precision. Moreover, learners can actively engage in providing feedback on educational practices, thereby positioning themselves as genuine agents of digital transformation.

### 7.4 Digital transformation of teaching models

In 1995 Japanese scholars Ikujiro Nonaka and Hirotaka Takeuchi proposed the SECI model, also known as the Knowledge Spiral Model. This theory describes the dynamic process by which knowledge is continuously transformed, shared, and created through four stages: Socialization, Externalization, Combination, and Internalization (Nonaka and Hirotaka, 1995). The model emphasizes the integration of multiple sensory modalities—such as images, sounds, language, actions, and text—sharing similarities with the multimodal teaching model proposed by Stein (2000). With the advent of digital and data technologies, particularly MOOCs and similar platforms in higher education, a digital SECI-based multimodal teaching model has the potential to significantly advance the digital transformation of teaching models in higher education. Scholars have explored various adaptations of the SECI model in digital education, examining its integration into course design and delivery, such as in Innovation and Digitization Management and Data-Based Decision Making, to enhance knowledge transfer (Żatuchin, 2024). Some propose a blended knowledge-sharing model based on the SECI framework (Hu et al., 2023), specifically addressing its application in design education and offering new theoretical insights. Additionally, a conceptual study (Rani, 2025) investigates the integration of IT adaptation with the SECI knowledge conversion process, proposing a model to improve program design in higher education.

The digital SECI-based multimodal teaching model involves four stages, forming a teaching loop of interaction, feedback, evaluation and testing (Zhang et al., 2024). The four stages are Interaction (Socialization), Feedback (Externalization), Evaluation (Combination), Testing (Internalization).

#### 7.4.1 Interaction (Socialization)

It is a phase that involves students’ autonomous learning and interactive discussions facilitated by digital platforms. Teachers preassign learning tasks through digital tools, allowing students to engage with resources such as audio, images and charts. Students then participate in group discussions to address key and challenging concepts which promote collaborative socialization.

#### 7.4.2 Feedback (Externalization)

Feedback involves teachers issuing simple tasks or surveys to evaluate students’ independent learning outcomes. By collecting and analyzing quantitative data from these tasks, teachers externalize knowledge by presenting results and identifying key takeaways for classroom instruction.

#### 7.4.3 Evaluation (Combination)

This phase integrates and synthesizes diverse sources of knowledge to generate new insights or information. In digital classrooms, teachers assess pre-class tasks and learning outcomes, highlighting areas of difficulty and guiding students to derive correct solutions. This stage emphasizes the use of oral explanations, gestures, and digital media to facilitate the transition from concrete to abstract knowledge, fostering epiphanies in students’ learning processes.

#### 7.4.4 Testing (Internalization)

Testing involves teachers assigning practice exercises to evaluate learning progress. By comparing pre-class task outcomes to in-class test results, teachers can assess the effectiveness of pre-class preparation and refine their instructional focus. Through this process, students achieve a cyclical transformation between tacit and explicit knowledge, internalizing professional knowledge and skills for application in real-world problem-solving.

The interactive SECI teaching model effectively converts explicit knowledge into students’ personal tacit understanding, enabling the practical application of professional skills while fostering reflection on their learning processes. This model enhances long-term learning retention and habits, improves cognitive and analytical skills, and supports personalized learning by tailoring instruction to individual learners. Simultaneously, it provides teachers with a broader array of resources and methodologies, driving professional growth and enriching the teaching experience.

### 7.5 Digital transformation of teaching scenarios

The digital transformation of teaching scenarios involves two primary aspects: The integration of physical teaching environments with digital technologies and the development of digital platforms.

Integration of physical teaching environments with digital technologies. Currently, both traditional and multimedia classrooms remain confined to single locations and isolated teaching scenarios. This segmentation of teaching processes across different settings hinders instructional interaction and makes it challenging to track the students’ learning progress. Digital technologies, however, can entirely remove the physical boundaries of teaching environments. The digital transformation of physical teaching spaces emphasizes the application of next-generation information technologies such as 5G, the Internet of Things (IoT), big data, cloud computing, and artificial intelligence (AI). This transformation entails optimizing and upgrading infrastructure, hardware, network conditions, and intelligent tools while continuing the development of smart campuses, smart classrooms, and smart living spaces. These advancements aim to create digital learning spaces that deeply integrate time, space, and teaching activities, blending offline and online learning in both virtual and physical environments (Goodyear et al., 2021). The goal is to promote scenario-based, experiential, and immersive teaching while supporting ubiquitous, blended, customized and immersive learning systems. Such systems enable students to make personalized learning choices based on their own pace and interests.

Development of digital platforms. A critical aspect of higher education’s digital transformation lies in technological innovation and platform construction. Emphasis is placed on enhancing students’ practical skills and experiential learning while establishing comprehensive technological system architectures and stable network infrastructures. Key areas include advancing AI, extended reality (XR), augmented reality (AR), and virtual reality (VR) technologies (Zhang, 2024). Digital transformation in higher education must sustain the traditional advantages of education dissemination while building comprehensive digital platforms encompassing centralized teaching and research, management services, and open forums. Interconnectivity and interaction among these platforms are essential to facilitate the extensive mining and integration of educational data. By leveraging learning analytics and educational data mining, it becomes possible to better align teaching services with learning needs, enabling precise content delivery and improving teaching quality and efficiency. The ability to seamlessly transition between multiple platforms and scenarios ensures the full integration of online and offline educational activities. This approach provides broader opportunities for personalized and targeted learning, allowing learners to maximize the use of educational resources from across society. It ensures equitable access to suitable educational resources based on individual needs, ultimately fostering education fairness and promoting lifelong learning and sustainable development.

### 7.6 Digital transformation of evaluation methods in higher education

The digital transformation of evaluation in higher education is not about the mere use of technology for its own sake, nor is it about maximizing technological application indiscriminately. It must not turn technology into a surveillance mechanism for teachers and students. Instead, digital technologies should be leveraged to establish a diversified and process-oriented evaluation system, optimizing evaluation processes through data analysis techniques.

Diversifying evaluation standards and driving innovation in teaching evaluation. Scientific evaluation standards are crucial tools for improving teaching quality. Higher education must move beyond single-dimensional talent evaluation models to develop diverse criteria that emphasize comprehensive ability assessments and highlight the role of evaluation in fostering talent development. By incorporating technology-supported evaluation tools and methods, such as automated scoring systems for online tests, more timely, accurate, efficient, and comprehensive feedback can be provided. These approaches also strengthen the students’ abilities for self-assessment and reflection, cultivating their awareness and capability to actively participate in the evaluation process.

Promoting digital literacy and establishing flexible, human-machine collaborative evaluation systems. The ultimate aim of evaluation is to improve teaching and enhance student learning. The process should focus on advancing the digital literacy of both educators and learners during the diverse teaching practices. Each participant acts as both an evaluator and an evaluatee. The advancement of digital technologies has made human-machine collaborative evaluation a viable approach to address diversity and personalization in higher education. Efforts should be made to encourage active participation from a variety of evaluative entities and to explore effective models of human-machine collaboration. This could include implementing digital certifications, promoting inter-institutional course selection, aligning standards, recognizing credits and fostering mutual trust in evaluation processes. By integrating internal and external data for associative analysis and multi-level calibration, a comprehensive lifelong learning system can be built.

Supporting teaching and learning practices with comprehensive, continuous data collection. The digital transformation in higher education evaluation needs to transition from isolated performance records to panoramic data collection. This involves dynamic analysis of learners’ processes across domains, including academic achievement, teaching effectiveness and social participation through the utilization of big data. Smart terminals, wearable devices, and intelligent education platforms can be employed to facilitate contextualized data collection. In addition to gathering text-based data, multimodal data such as audio, video, psychological indicators and physiological signals can also be captured, providing a holistic view of critical moments and typical behaviors in students’ development. These insights focus on academic performance, physical and mental well-being as well as social practice to ultimately contribute to a widely agreed-upon and inclusive theoretical framework for evaluation indicators. This comprehensive approach enables a full-spectrum, continuous evaluation system that aligns with the diverse and evolving needs of higher education. Thus, fostering equitable and effective teaching and learning outcomes.

## 8 Conclusions and perspectives

The digital transformation in higher education is a need that represents a dynamic process driven by the deep integration of digital technologies with educational practices. Our study focuses on enhancing the digital literacy of faculty and students, transforming educational philosophies, optimizing teaching models, integrating educational resources, and reforming evaluation systems. Emphasis is placed not only on achieving a profound integration of digital technologies within higher education process but also on fostering the symbiotic coexistence and coordinated evolution of technological and educational ecosystems.

As for the theoretical basis, this article derives the connotation of digital transformation in higher education through literature analysis. In the following exploration, first, illustrative case studies from three universities—Havard University, Tsinghua University, and the University of Melbourne, were conducted and a triple framework for digital transformation in higher education consistent of value logic, technology logic, and practice logic was proposed, with a comparison to other models. Second, four practical dilemmas confronting the digital transformation in higher education that occur in the current state were analysed, including the psychological challenges for university faculty and students as well as the physical dilemmas for the teaching and feedback process. Finally, six actionable pathways for addressing these challenges were put forward. The key highlight of this research is the construction of the teaching model innovation of digital transformation in higher education, which is the SECI teaching model based on digital technology.

The implications of this study extend beyond theoretical contribution and offer actionable insights for a spectrum of stakeholders engaged in the digital transformation of higher education. For policy makers and institutional leaders, the triple-logic framework provides a strategic diagnostic tool to formulate holistic policies and investment strategies that ensure technological adoption is fundamentally guided by pedagogical value and supported by robust digital literacy programs. Consequently, university administrators and instructional designers can leverage the six implementation pathways as a practical blueprint to prioritize initiatives, allocate resources effectively, and navigate the complex process of systemic change. Concurrently, the study equips educators with a validated conceptual model—exemplified by the SECI-based teaching approach—to redesign pedagogical practices, transition their professional role from knowledge deliverer to learning facilitator, and confidently adopt digital tools that promote interactive and personalized learning. Furthermore, by delineating clear operational dilemmas and transitional pathways, this research provides researchers with a robust foundation for further empirical inquiry into the dynamics of digital transformation. Collectively, these insights underscore the study’s practical value in guiding a coordinated, multi-stakeholder effort towards a more sustainable and impactful digital future for higher education.

It is worth indicating the existence of some limitations and, therefore, further required steps for this study should be mentioned in this context. This paper represents a systematic conceptual-theoretical exploration of the digital transformation in higher education. Future research directions will delve deeper into specific subtopics, such as refining implementation approaches from the perspectives of students, policy, and employment demands and capabilities. Additionally, the developed meso- and micro-level frameworks will undergo Delphi validation to enhance their relevance and utility in a more rigorous and direct manner.

Finally, the use of such technological tools and internet-related options comes with risks in terms of cybersecurity, data protection and vulnerable to hacking that should be always taken into consideration for the protection of the technology-based education systems to optimize its utilization and make them safe spaces for all the users. This means that developing such teaching models require well-trained experts, engineers and technicians within the education institutions as well. Creating safe systems will further encourage both learners and educators to get involved with confidence.

## Data Availability

The original contributions presented in the study are included in the article/supplementary material, further inquiries can be directed to the corresponding author.
